# Sources of Indoor Air Pollution and Respiratory Health in Preschool Children

**DOI:** 10.1155/2009/727516

**Published:** 2010-02-03

**Authors:** Virginia Fuentes-Leonarte, Ferran Ballester, José Maria Tenías

**Affiliations:** ^1^Department of Health and Environment, Centre for Public Health Research (CSISP), 46020 Valencia, Spain; ^2^CIBER Epidemiology and Public Health (CIBERESP), 8003 Barcelona, Spain; ^3^Research Support Unit, Hospital La Mancha Centro, 13600 Alcázar de San Juan, Spain

## Abstract

We carried out bibliographic searches in PubMed and Embase.com for the period from 1996 to 2008 with the aim of reviewing the scientific literature on the relationship between various sources of indoor air pollution and the respiratory health of children under the age of five. Those studies that included adjusted correlation measurements for the most important confounding variables and which had an adequate population size were considered to be more relevant. The results concerning the relationship between gas energy sources and children's respiratory health were heterogeneous. Indoor air pollution from biomass combustion in the poorest countries was found to be an important risk factor for lower respiratory tract infections. Solvents involved in redecorating, DYI work, painting, and so forth, were found to be related to an increased risk for general respiratory problems. The distribution of papers depending on the pollution source showed a clear relationship with life-style and the level of development.

## 1. Introduction

In our society, most people spend the majority of their time indoors, at home, work, or school, in public buildings, and so forth. For this reason, the quality of indoor air affects the quality of life and the well-being of the population in general, with the exposure to diverse indoor pollutants presenting a higher risk for developing various diseases such as those affecting the respiratory system [1]. 

Young children constitute a special risk group due to the fact that their respiratory system is not yet completely developed until the age of six. Thus, for example, while the number of alveoli at birth is approximately 24 million, by the age of four, this number increases to 250 million [2]. Furthermore, children breathe more air in proportion to their weight than adults and, in the case of very young children, they spend proportionally more time indoors. For all these reasons, the first years of life constitute a critical period for an individual's exposure to air pollution. 

The principal sources of indoor air pollution are linked to the methods used to heat the home as well as to the domestic activities related to the preparation of food (cooking). Other habitual pollution sources include the use of tobacco and of chemical products used for cleaning, building, and decorating [3].

In developing countries, the population depends on wood, coal, or biomass fuels to satisfy their basic energy needs [4]. Cooking and/or heating with these energy sources, in many cases over open fires and with no chimney or exhaust system, leads to significant concentrations of indoor air pollution, including both gases and particles in suspension. In poorly ventilated houses in which this type of fuel is used, indoor pollution levels can reach concentrations up to 100 times greater than the currently established limits for outdoor air pollution. This type of exposure is particularly pronounced in women and children, who spend most of their time indoors and who dedicate more time to domestic chores.

According to the WHO [5], in the poorest countries up to 5% of the total amount of disease can be attributed to the use of solid fuels as energy sources. This percentage is in large part due to the impact of indoor air pollution on deaths caused by acute respiratory infections (ARIs), which cause 19% of deaths in children under the age of five [6].

In the developed world, domestic combustion sources tend to be cleaner, using fuel sources such as gas and electricity. In these countries, problems of indoor air pollution tend to be related to other compounds such as nitrogen dioxide, derivatives from tobacco smoke, volatile organic compounds (VOCs), and other chemical compounds [3]. In addition, in order to save energy and maintain a constant indoor temperature, the windows of the buildings are rarely opened; indeed, there is a trend to design buildings with ever greater amounts of insulation. For these reasons, the renovation of the indoor air diminishes and, because the channelling of pollution generated by indoor sources to the exterior of the building is increasingly avoided, the concentration of indoor air pollution increases correspondingly.

In a previous paper [7] we reviewed articles published on indoor air pollution levels and the respiratory health of children. However, from the point of view of prevention, it may be more useful to have at our disposal evidence related to the sources of the exposure, such as the types of domestic appliances that emit gases, the burning of fuel in the home, or the use of chemical products that emit gases in building interiors. The identification of those sources most related to the effects on health in young children would allow for the establishment of strategies to reduce exposure, and, as a consequence, health problems in this subset of the population. Our objective is thus to review the scientific literature on the relationship between various sources of indoor air pollution and the respiratory health of children under the age of five.

## 2. Materials and Methods

The bibliographic searches were carried out with PubMed (National Library of Medicine and National Institute of Health) and Embase.com (Elsevier). The period chosen was from the years 1996 to 2008, both inclusive. No restrictive criteria were set concerning the language of publication. 

We chose to undertake the most exhaustive search possible. As such, the broadest descriptors were used to identify those articles dedicated to respiratory illness and indoor air pollution (Table 1). The limit for the age of the subjects was set at under five years of age.

We looked for studies in which correlation measures were established between one or more indoor pollution sources and at least one indicator of respiratory health in children under five. Those studies in which the only source of pollution examined was the passive exposure to tobacco smoke were not included in the present review because the sheer volume of publications on this topic merits separate consideration. A major selection criterion was that the source(s) of indoor air pollution was clearly identified in the published work. So, studies in which the pollutants are measured as levels but without a source of pollution identified were excluded. In case of doubt, the entire text was read. 

We then proceeded to extract and classify the information using a table agreed upon through consensus in which the following items were used: country (distinguishing between developed and developing countries), type of study, author and year of publication, and population data, which included age of the subjects and number of cases. With regard to the health indicators, the items were as follows: definition of the incidence and information source (questionnaire, interview, clinical visit, etc.). With regard to exposure, we classified the pollution source. We also collected the adjusted correlation measurements and the interval of confidence 95% values as well as the adjustment variables that were examined. The possibility of carrying out a meta-analysis was considered taking into account the criteria that more than two studies analyzing the impact of the same type of pollution source on the same health indicator in comparable populations were found.

The results of those studies which provided adjusted correlation values for the most important confounding variables were considered to be the most relevant. These variables included sociodemographic data, parents' background, personal background, and especially the presence of other pollutants, both indoors and out. In addition, those studies with an adequate sample size which used a cohort or a case-control design within a cohort were considered to be of greater relevance, as were those in which the case definition was clear and rigorous. Each study was critically appraised identifying possible threats to its internal validity. 

## 3. Results and Discussion

### 3.1. Results

A total of 332 documents were obtained from a first reading of the abstracts: 143 were found exclusively on PubMed and 123 on EMBASE, with 66 studies appearing in both data bases (19.9%). Finally, 25 publications were considered to be relevant in accordance with the aforementioned criteria. 

#### 3.1.1. Studies Analyzing the Relationship between Gas-Powered Indoor Pollution Sources and Respiratory Problems (See Table 2)

Nine studies which met the selection criteria were identified; five were cohort studies, two had a transversal design, and two were case-control studies, one of which took place within a cohort.

Five of the studies were carried out in the United States, two in Europe (Sweden, Spain, and the United Kingdom), and two in Africa (Zimbabwe and South Africa).

**Studies Carried Out in the U.S.**

Using a cohort of 936 children living in Tucson, Arizona, Aldous et al. [8] studied the development of lower respiratory tract illnesses (LRTIs) during the first year of life. The authors took various domestic exposure factors into consideration, including the energy sources used for heating, refrigeration, and cooking. They observed an increase in LRTIs in those children who lived in houses with cooling systems based on the evaporation of a cold liquid into the indoor air: OR 1.8 (IC 95% 1.1–3.0) for LRTIs involving difficulties breathing (wheezing), and OR 1.5 (IC 95% 0.7–4.0) for LRTIs without breathing difficulties. The authors attributed this increase in risk to a greater exposure to molds, mites, and pollen coming from the cooling system. Gas heating was also linked to an increase in risk, especially for LRTIs with wheezing. In addition, the authors noted a positive and significant tendency related to the perceived lack of cleanliness in the neighborhood.

In Connecticut and Virginia, Triche et al. [9] followed a cohort of children up to their first birthdays, noting episodes of coughing and wheezing. The authors linked the incidence of both symptoms to the type of fuel used for heating, finding a positive and significant relationship between the symptoms observed and gas heating, for example (RR 1.25; IC 95% 1.05–1.50). The estimates were adjusted for various types of confounding variables; moreover, the homes of smokers were excluded from the study.

In their study conducted in Connecticut and Massachusetts, Belanger et al. [10] recruited a cohort of children with a high risk for developing asthma (all had one asthmatic sibling) and followed them during their first year of life. Through questionnaires, the mothers recorded the appearance of respiratory symptoms (persistent wheezing and cough). The authors analyzed the indoor exposure by typifying the use of gas or wood as energy sources together with monitoring levels of NO_2_ and different allergens. The use of gas heaters was correlated in a statistically significant way to the incidence of persistent cough in children of mothers with no personal history of asthma. A similar correlation was found when the energy source was substituted for NO_2_ concentrations, with a 21% increase in the frequency of persistent cough for each 10 ppb increase in NO_2_ levels (IC 95% 5–40%).

These same authors later conducted a transversal study with a different cohort [11] to examine the relationship between the use of gas energy sources (versus other types of fuel) and respiratory problems. They observed a significant correlation between wheezing and intercostals muscle retractions in children who lived in multifamily housing. When they replaced the gas source with NO_2_ levels in the regression models, they found a significant relationship with both symptoms. They concluded that this compound may explain in part the relationship between gas energy sources and these symptoms.

**Studies Conducted in Europe**

In a cohort of children born in Stockholm, Emenius et al. [12] carried out a nested case-control study, conducted when the children were between 1 and 2 years of age, in which the relationship between exposure to various types of domestic pollution and the incidence of persistent wheezing (3 or more episodes unrelated to the common cold from the age of 3 months together with symptoms of bronchial hyperreactivity such as coughing while sleeping, playing, or laughing) was evaluated. In the multivariate analysis, the authors observed a higher risk in children who lived in homes with gas cooking ranges (OR 1.72; IC 95% 0.77–3.87), although few homes (8.5%) actually cooked with gas. Increases in indoor NO_2_ levels were not associated with the aforementioned symptoms in a significant fashion (OR 1.06; IC 95% 0.74–1.52), although there was a significant relationship between the symptoms and outdoor NO_2_ levels (RR 1.73; IC 95% 1.05–2.82).

In three cohorts of children born in Spain (Barcelona and Menorca) and the United Kingdom (Ashford), Sunyer et al. [13] studied the relationship between indoor NO_2_ levels and the incidence of lower respiratory tract infections (LRTIs) in the first year of life. The results showed a lack of significant correlations between NO_2_ and LRTIs. Moreover, neither the use of gas cooking ranges or gas heating was associated with significant variations in the occurrence of LRTIs. In a previous study, [25] the authors had observed a close relationship between NO_2_ levels and the use of gas energy sources.

**Studies Conducted in Africa and Asia**

Kristensen and Olsen [16] followed a cohort of 571 children born in Soweto (South Africa) during the first year of life, noting the appearance of episodes of acute respiratory infections (ARIs). They did not find a significant association between the incidences of such episodes with the use of different cooking fuels.

In a sample representative of 3559 children under the age of five taken from a national health survey in Zimbabwe, Mishra [14] identified those cases with symptoms compatible with an ARI in the two weeks prior to the survey. They collected information about the type of cooking fuel used, classifying different types of fuels as follows: highly pollutant (wood, straw, dung), mildly pollutant (kerosene and coal), and least pollutant (gas and electricity). After adjusting for different variables, they found that the use of highly pollutant cooking fuels doubled the risk for respiratory infections. Other factors associated with this problem were the age of the child, with the most crucial period being between 6 and 11 months, and the area of residence.

In a case-control study, Singh et al. [15] analyzed various factors associated with persistent and/or recurrent cough in children between the ages of 1 and 15 living in the area of Ludhiana, in Northern India. Among other factors, the authors analyzed the relationship between this health problem and the use of smoke-producing fuels: kerosene, wood, cow dung, and so forth. They found a nonsignificant increase in the incidence of the symptoms and the use of these energy sources (unadjusted OR 1.49; IC 95% 0.61–3.66).

#### 3.1.2. Studies Analyzing the Relationship between Indoor Pollution from the Use of Solid Fuels, Biomass Fuels, Oil, and Kerosene with Respiratory Symptoms (Table 3)

We found twelve studies dealing with this issue. The majority of them (seven) were carried out in communities in the developing world in which the population uses highly pollutant materials (biomass and coal) and inefficient instruments to meet their daily energy needs. Six of the studies had a case-control design, four used cohorts, and one was a transversal study.

**Studies Conducted in Africa and Asia**

In an exploratory study, Sharma et al. [17] examined two suburbs of Delhi. The authors compared the incidence of LRTIs in children under the age of four divided into two groups: those that lived in homes in which kerosene was used to cook and those in which wood was used as the main cooking fuel. No significant differences were observed, probably because the kerosene group showed a greater tendency to cook indoors in the presence of the children [17].

In a case-control study carried out in India by Broor et al. [18] with children under 5 to examine the relationship between the use of fuels other than liquid petroleum gas (LPG) and LRTIs, 36.8% of the cases and 20.6% of the controls used a fuel other than LPG. The authors observed a significant correlation between the incidence of LRTIs and the use of non-LPG cooking fuels: OR = 2.51; IC 95% 1.51–4.16 [18]. Another case-control study conducted in Calcutta by Mahalanabis et al. (2002) in children between the ages of 2 and 35 months showed a clear correlation between the use of solid fuels and hospital admissions for pneumonia (OR: 3.97; IC 95% 2.00–7.88) [19].

As commented above in the section on studies related to energy sources, Mishra (2003) [14] carried out a study in Zimbabwe using the *Fourth Zimbabwe Demographic and Health Survey* (ZDHS) to examine the relationship between acute respiratory infections, defined as the presence of cough and tachypnea in the two weeks prior to the survey in children under the age of five. The authors found that 66% of the children lived in homes in which highly pollutant fuels (wood, dung, straw) were used and that there was a significant correlation between the use of these fuels and the incidence of ARIs (OR: 2.20; IC 95% 1.16–4.19) with respect to those who lived in homes in which cleaner fuels were used [19]. In a similar study carried out in India (2005) with children between 0 and 35 months of age, upon controlling for potentially confounding variables such as passive exposure to tobacco smoke (PETS), the authors found a positive, albeit slightly lower, correlation (OR: 1.41; IC 95% 1.17–1.70) [26].

In the suburbs of Bangladesh (Dacca), Khalequzzaman et al. (2007) conducted a case-control study with children under 5. They evaluated the presence of respiratory symptoms and signs as well as respiratory disease (bronchiolitis, asthma, pneumonia) depending on the use of solid biomass fuels. In homes in which biomass was used as fuel, clear associations were found with shortness of breath (OR: 6.30; IC 95% 1.60–29.1), intercostals muscle retraction (OR: 5.50; IC 95%1.30–29.40), and wheezing (OR: 4 IC 95% 1.10–16.20) [20].

**Studies Conducted in South America**

In their study on a group of 20 children under the age of 2 along with 36 mothers, all residents of the state of Chiapas (Mexico), Riojas-Rodríguez et al. (2001) observed a protective, albeit nonsignificant, effect (RR = 0.28; IC 95% 0.07–1.18) related to the use of closed “Ceta”-type ovens as opposed to the use of open fires for heating and cooking. This is due to the fact that the emission of particles in suspension—principally PM10 particles—is higher in the latter. This study was not included in the summary table as its principal objective was to study the correlation between respiratory symptoms and the levels of PM10 particles in suspension and the number of cases was very low [33].

In a transversal study carried out by Schei et al. (2004) in Guatemala with 1058 children between the ages of 4 and 6, the authors used the ISAAC questionnaire to examine the presence of asthma symptoms (wheezing, night cough) in relationship to the cooking methods used in the home. In 51.5% of the homes, an oven with an exhaust chimney was used while gas and open fires were used in only in 10.4% of the homes. In 38.1%, however, open fires were used. The authors observed a statistically significant relationship between the incidence of wheezing and the use of open fires for cooking [21].

**Studies Conducted in the United States**

In 1996, Robin et al. conducted a case-control study pairing hospital admissions for LRTIs in Navajo children between 1 and 24 months of age and the use of wood-burning stoves. They found an excess risk more than five times greater in those children who lived in houses in which wood was used as a cooking fuel, although with an ample interval of confidence given the low number of subjects in this group (8.9% of the cases). The magnitude of correlation diminished less than 20% after adjusting for potentially confounding variables [22].

In the cohort study conducted in Connecticut by Triche et al. (2002) [9], mentioned above in the section on gas energy sources, the incidence of wheezing and cough was evaluated during the first year of life. The number of total days of coughing was correlated to the use of wood-burning stoves (OR = 1.10; IC 95% 1.02–1.19), while the number of episodes of coughing was related to the use of kerosene heaters (1.07; IC 95% 1.00–1.15). The number of total days of wheezing, on the other hand, was associated with the use of gas heaters (1.25; IC 95% 1.05–1.50).

**Studies Conducted in Europe**

In a cohort study, in this case carried out retrospectively, conducted in Finland (2001), the authors identified living on a farm between the ages of 0 to 6 years as a protective factor against allergic rhinoconjunctivitis with an OR of 0.51 (0.5–0.75, *P* < .0001). In this sample population, living on a farm was frequent for subjects whose houses were heated with wood stoves [23].

In Teplice (Czech Republic), a cohort of 452 children was followed up to the age of 3. The authors analyzed the correlation between exposure to various types of domestic pollutants with the incidence of LRTIs (excluding pneumonia). After adjusting for different covariables, the authors found a significant increase in the incidence of LRTIs in those children living in coal-heated. This correlation was higher still if the child had not been breastfed (RR 2.77; IC 95% 1.45–5.27). Other energy sources such as natural gas or propane were not significantly linked to the incidence of LRTIs [24].

#### 3.1.3. Studies Analyzing the Relationship between Sources of Indoor Pollution from Chemical Products and Respiratory Problems (Table 4)

Six of the studies analyzed the relationship between sources of chemical compounds in the home and the respiratory health of young children. All of these studies were conducted in Northern European countries and all but one were conducted within the framework of various birth cohorts.

In the Oslo (Norway) birth cohort, various nested case-control studies were carried out. In the first one, Jaakkola et al. [27] examined 251 case-control pairings to determine the relationship between bronchial obstruction during the first years of life and the presence of polyvinyl chloride (PVC) in the plastics, carpeting, and other wall and floor coverings in one or more rooms of the home. The authors found significant correlations between bronchial obstruction and the use of PVC as a flooring material or floor covering (OR: 1.89; IC 95%: 1.14–3.14) as opposed to wood or parquet. They also found a relation that was just below significance between this particular respiratory symptom and the use of wallpaper (OR: 1.58; IC 95%: 0.98–2.54) as compared to painted walls.

Another study of the same cohort [28] used a larger sample (304 case-control pairs) to analyze the impact of using either paper or plastic wallpaper. The novelty of this study was that the possibly modifying role of ventilation was evaluated, although for this particular factor, measurements were carried out for only 172 pairs. The results for the entire sample confirmed those of the afore-mentioned study [27]. The authors also found that the impact was greater on children who lived in homes with poor ventilation (37% of all the homes in the sample).

One of the researchers from the previous studies, Jaakkola et al., also conducted a study on the effect of plastic wall and floor coverings on the child population of Finland [29]. Within the framework of a populational transversal design, the author analyzed the relationship between exposure to said materials and the incidence of illnesses (such as asthma) and symptoms, including wheezing or persistent cough, in 2568 children between the ages of 1 and 7. The results showed that the emissions of these materials within the home could have adverse effects on the respiratory health of children, with significant correlations between the presence of these materials and persistent wheezing, cough, and phlegm.

Diez et al. conducted two studies on the correlation between redecorating activities and respiratory effects in children with allergy risk factors in the Leipzig (Germany) cohort LARS [30, 31]. The first study [30] evaluated the influence of the exposure to products used for redecorating the home during pregnancy on respiratory problems in the child's first year of life. In the majority of cases, children of parents who had undertaken activities such as painting or changing the carpeting presented a higher risk of suffering pulmonary infections during the first 6 weeks of life (OR: 5.60; IC 95% 1.30–24.00) and also of having episodes of wheezing during the first year (OR: 1.90; IC 95% 1.10–3.50). The authors also measured the indoor levels of several volatile organic compounds and found the highest styrene levels in homes in which new wall-to-wall carpeting had been installed. Furthermore, upon studying the relationship between these compounds and health problems, they observed a correlation between respiratory infections at 6 weeks and living in houses with styrene levels >2.0 *μ*g/m^3^ and benzene levels >5.6 *μ*g/m^3^ (OR: 2.10 (1.10–4.20) and 2.40 (1.30–4.50), resp.). Nevertheless, the authors did not provide any results concerning the possible correlation between volatile organic compounds and wheezing in the first year of life.

In their second study using the LARS cohort, Diez et al. [31] followed a subset of 186 of the aforementioned children up to the age of two, collecting information about decorating activity in the home and respiratory symptoms. The results indicate that decorating activities have an important influence on the appearance of obstructive bronchitis during the first and second years of life, with statistically significant ORs greater than 4. The effect of such activity on the incidence of wheezing was lower, but also significant (OR: 3.00; IC 95% 1.00–9.10).

Finally, Emenius et al. [32] also studied the correlation between having painted an area within the house (or, specifically, the child's room), ventilation, and the incidence of recurrent wheezing up until the second year of life. The authors found a relationship between having recently painted the child's room and recurrent wheezing (OR: 1.70; IC 95% 1.30–2.60). This correlation did not change when adjusted for the amount of ventilation in the home.

### 3.2. Discussion

In the studies analyzing the correlation between the use of gas energy sources and respiratory health in young children, the results vary for the most part depending on the geographic/cultural context in which the studies were carried out. In most of the studies conducted in developed countries, gas energy sources were associated with an increase in various indicators of respiratory health, especially symptoms of bronchial obstruction/asthma (coughing, wheezing), but not necessarily respiratory infections. In these studies the energy source used as a reference was one that was supposed to pollute less, such as electricity. However, in the developing countries gas energy sources formed part of the reference category against which the impacts of more highly pollutant energy sources such as wood, coal, or animal waste were compared. In this context, one can safely say that the substitution of these types of fuel with a gas energy source would lead to improvements in the indicators analyzed.

Solid fuels constitute the principal source of energy in the homes of nearly 3 billion people. Their use is clearly linked to poverty, a condition that implies poor access to a healthcare system. The results of the studies carried out to date along with various other evaluations of the impact of air pollution from the use of biomass on health in the world indicate that indoor pollution in the poorer countries is one of the principal causes of death in the world [34, 35]. For this reason, the evaluation and application of effective intervention strategies should form a part of the policy priorities for public health at the global level [36, 37]. Use of this type of fuel has also been documented in the Scandinavian countries, mostly due to the use of wood-burning stoves for heating. However, factors such as living near a farm, most likely along with other variables, act as confounders, leading to the idea that this type of fuel can act as a protective factor against allergy problems such as asthma [23, 38]. 

Recently, various studies conducted in northern Europe have shown that there is a group of sources of chemical compounds found in the home that are related to increased risk of respiratory diseases [27–31]. The authors of both the Norwegian cohort study [27, 28] and the Finnish transversal [29] study found a relationship between respiratory problems in children when PVC or other plastics were used as wall coverings in the home. One group of chemical compounds used in the manufacture of plastics is that of the phthalates. These compounds can be released into the indoor atmosphere of the home and become incorporated into the organism of the people thus exposed [36]. Indeed, various recent studies have evaluated the relationship between these compounds and allergic and respiratory problems in children.

Bornehag et al. [39] studied the possible correlations between the concentration of various phthalates in house dust and allergic symptoms in a study using 200 case-control pairs nested in a cohort of children in Sweden. The results indicate that there is an association between certain phthalates and rhinitis, eczema, and asthma, even when the phthalate levels fall within the normal range for these compounds in Sweden. Kolarik et al. [40] analyzed the same relationship in 102 children between the ages of 2 and 7 who had exhibited allergic symptoms in the past 12 months and 80 controls living in Sofia and Burgas (Bulgaria). The results indicated a significant correlation between several of the phthalates analyzed and the incidence of wheezing.

In the other three studies included in this review, the exposure factor studied was the case in which decorating and/or construction work had been carried out at home, especially the recent painting of the child's room or the installation of wall-to-wall carpeting [30, 31, 41]. In a recent review [39], Mendell found that aliphatic compounds were correlated to painting in the home and, what is more, that these compounds were associated with immunological changes. In addition, certain types of wall-to-wall carpeting used in Germany have been found to be related to the emission of volatile organic compounds.

## 4. Conclusions

The distribution by country of the studies examining various pollution sources reveals a clear relationship between life-style habits and the degree of development. For example, most of the studies on gas (NO_2_) sources were conducted in Western countries, with only two being carried out in the developing world. In contrast, studies concerned with biomass fuels were mostly carried out in developing nations. With respect to indoor pollution due to sources that emit chemical compounds, the only studies found were conducted in Northern Europe.

The correlation between wood fuel and respiratory problems is clearly documented in the developing world. For example, according to a recent, exhaustive study carried out by the World Health Organization [38], the risk of young children (under 5 years of age) contracting pneumonia has been observed to increase by 80% when they are exposed to the combustion of solid, unprocessed fuels. 

The principal pollutant of gas energy sources is NO_2_. In those studies in which the role of this compound was also included, the general tendency was that its effect was similar, both in direction and magnitude, to that assigned to gas energy sources in general. Taking this into account, we can hypothesize that the effect observed for the gas energy sources is probably mediated by the action of NO_2_. This remains uncertain, however, since none of the studies introduced both the indicator of the energy source and the NO_2_ levels in the same multivariate model.

All of the studies about chemical compounds found in home and respiratory health of children have in common the fact that they were carried out in cold areas in which there is less ventilation of the home and where the uses of wall-to-wall carpeting and insulation are frequent. All the studies observed significant correlations between the presence of these materials in the home and respiratory problems in children.

The European Union has yet to establish guidelines for indoor pollutants, probably due to the absence of a systematic study of the risks that these contaminants pose for human health. According to the results of the THADE (“Towards Healthy Air in Dwellings in Europe”) study published in 2006, there is a lack of evidence-based information on the appropriate measures for preventing adverse effects on health caused by indoor pollution [42]. Also, for most of the pollutants, their effects on human health are highly debatable [43]. It would thus be beneficial to conduct studies determining these risks for the most vulnerable members of the population, including, for example, children.

In this review it was not possible to summarize the results of the studies through metanalysis due to the lack of a minimum number of studies with the same design, analyzing the same exposure source on the same result indicator within comparable populations. Likewise, we cannot rule out a certain bias in the publications chosen, which could obviate the results of other studies that we were unable to identify with our search strategies and criteria. Nevertheless, it is worth noting that the strategy used was exhaustive in nature and included the revision of references included in the bibliographies of the studies thus selected.

The use of pollution sources as an exposure variable could be a disadvantage with respect to the study of the impact of the concentrations of individual pollutants, especially when establishing norms and regulations for appropriate levels of these substances. There are, however, advantages to this strategy in that it allows for the analysis of a given exposure as a complex mixture of pollutants which, if studied individually, could lead to confusion bias due to correlations between the various components. Furthermore, it is much simpler to monitor and translate the results of such studies into preventative measures to be divulged either to the general population or to certain risk groups in order to decrease their exposure to pollution within their own homes.

## Figures and Tables

**Table 1:** Descriptors used in the bibliographic searches.

	PubMed	EMBASE (EMTREE)
Respiratory diseases	Respiratory Tract Diseases	Respiratory tract disease
Indoor air pollution	Air Pollution, Indoor	Indoor air pollution
Children	Infant, newborn	Newborn
	Infant	Infant
	Child, preschool	Preschool

**Table 2:** Studies analyzing the relationship between the use of gas in the home and the respiratory health in children under the age of five.

Location Year Author	Design	Sample population: Age Number of cases	Disease studied		Exposure			Control variables	Results: adjusted OR/RR (IC 95%)
			Case definition	Information source	Source, chemical products	Evaluation	Exposure (%)		
							
Studies conducted in the United States									

Tucson, USA 1996 Aldous, et al. [8]	Cohorts	936 children followed by pediatricians during the first year of life	Lower respiratory tract illnesses (LRTIs) Three indicators: - First episode of LRTI of any kind (303) - First episode of LRTI with wheezing (196) - First episode of LRTI without wheezing (60)	Pediatric practice. Identified using clinical criteria, including imaging techniques (Rx)	Heating (gas, electric, steam, other) Cooker (electric, gas, oil, other)	Q administered by a nurse to the parents	Central gas heating (64%), Gas heating through walls or floorboards (6%), other (30%) Cook range: Electric (61%) Gas (39%)	- Parents: educational level, ethnic group, history of hay fever or asthma - Perceived lack of cleanliness in the neighborhood, no. of rooms, shared rooms - Smoking - Pets (dogs, cats) - No. of siblings and other children - Child: sex, breastfeeding.	- OR LRTI gas heating (central or through walls/floorboards) versus other types of heating - LRTI with wheezing Central OR 1.2 (0.8–1.8) Walls/floorboards 1.6 (0.8–3.2) - LRTI without wheezing Central OR 1.2 (068–2.2) Walls/floorboards 1.6 (0.3–3.9)

Connecticut and Virginia 2002 Triche, et al. [9]	Cohorts	890 children followed for the first year of life	Episodes/Days of wheezing and episodes/days of cough during the winter months (October–April)	Interview and telephone call every 15 days	Gas heating	Q	2.8%	Sociodemographic factors - Allergies and/or asthma in the mother - Mother's educational level - Season of birth - No. of children - Breastfeeding	Gas heating: episodes with wheezing: 1.28 (0.99–1.67) Days with wheezing: 1.25 (1.05–1.50)

Connecticut and Massachusetts 2003 Belanger et al. [10]	Cohorts	849 newborns with an asthmatic sibling Followed for 1 year	Wheezing Persistent cough Days with symptoms during the first year of life: - None - <30 d - ≥30 d	Questionnaire mother/birth, 2–4, 6, 9, 12 months	Heating with gas, wood	Q	Gas: 34.2% Wood: 5.9%	- Sex - Ethnic group - Parent with history of asthma - Mother: allergies, smoking during pregnancy, educational level - Family income - Allergens (mites, cockroaches, cats, dogs) - Mold	OR Wheezing - Gas: 1.28 (0.88–1.86) - Wood: 0.76 (0.37–1.55) Persistent cough - Gas: 1.52 (1.06–2.18) - Wood: 1.68 (0.89–2.30)

Connecticut and Massachusetts (USA) 2006 Belanger et al. [11]	Transversal	728 asthmatic children <12 years (<6 years: 66.5%) with an asthmatic sibling	Presence of and days with respiratory symptoms (wheezing, persistent cough, dysnea and intercostals muscle retraction) in the month prior to the Q	Questionnaire (mother)	Gas sources: heating, dryers	Q	Heating: 54.6% Dryer: 7.4%	- Parent's education and ethnicity - Smoking, house characteristics, energy sources in the home - Child's age	Gas heating in multifamily houses: OR Wheezing: 2.27 (1.15–4.47) Cough: 1.19 (0.66–2.16) Dysnea: 2.38 (1.12–5.06) Intercostal muscle retraction: 4.34 (1.756–10.69)

Studies conducted in Europe									

Stockholm (2002) Emenius et al. [12]	Case-control nested in the BAMSE cohort	Children between 0 and 2 years of age Cases: 98 Controls: 195	Cases: persistent and/or recurrent wheezing Controls: residents in the same area	Questionnaire at 2 months and at 1 and 2 years	Gas cooking ranges	Q	8.5%	- Sex - Genetic history - Mother's age - Smoking in the mother - Breastfeeding - Age of building	Use of gas cooking range OR 1.72 (0.77–3.82)

Ashford (UK); Barcelona and Menorca 2004 Sunyer et al. [13]	Cohorts	642 children (Ashford) 487 children (Barcelona) 482 children (Menorca) Followed for the first year of life	Incidence of Lower Respiratory Tract Infections (LRTIs) in the first year of life: Ashford 39.6% Barcelona 28.4% Menorca 45.3% Use of antibiotics	Mothers' comments, clinical histories, and history of antibiotic prescriptions; Monthly telephone call (Barcelona)	Gas cooking range, heating	Q	Gas cooking ranges: 60.12–74.13% Gas heating: 24,69 a 83,49%	- Occupation of, allergies in, or atopy in parents - Smoking - Family size - Outdoor NO_2_ - Breastfeeding	OR gas cooking range: 0.91 (0.69–1.20) OR gas heater: 1,01 (0,75–1,36)

									
Studies conducted in Africa and Asia									

Zimbabwe 2003 Mishra et al. [14]	Transversal	3559 children <5 years of age	Acute respiratory infection (ARI) (coughing with dysnea and tachypnea in the two weeks prior to the survey): 16% ARI	Children selected from a national health survey (*Zimbabwe Demographic and Health Survey*, ZDHS)	Cooking fuel: - Highly pollutant (wood, straw, dung) - Mildly pollutant (kerosene, coal) - Least pollutant (gas, electricity)	Q	66% of children live in homes in which highly pollutant cooking fuels are used	- Mother: age and educational level - Socioeconomic level, residence (urban/rural), region, religion - Child: age, sex, birth order, and nutritional state	OR (IC 95%) for ARI: - Least pollutant fuels (ref) - Mildly pollutant fuels: 1.33 (0.64–2.77) - Highly pollutant fuels: 2.20 (1.16–4.19)

Ludhiana, India. 2002 Singh et al. [15]	Case-control	Cases: children between 1–15 years of age with persistent and/or recurrent cough (24) Controls: Randomly selected (500)	Persistent (>3 weeks) and/or recurrent (4 or more episode of over 10 days' duration) cough in one year	Questionnaire (parents)	Smoke-producing fuels	Q (parents)		- Parents: socioeconomic level, history of illness. - Smoking - Type of house (pucca, kutcha, mixed). - Hacinamient - Pets	Use of smoke-producing fuel versus other type of fuel OR (unadjusted): 1.49 (0.61–3.66) Adjusted correlation: *t* value = 1.668 (*P* = .095)

Soweto, South Africa 2006 Kristensen et al. [16]	Cohorts	Cohort of 571 children followed from birth throughout the first year of life.	Acute respiratory infection (ARI): 489 episodes Incidence: 1.56 per child per year	Home visits by health care workers	Cooking fuel: electricity or gas versus paraffin/coal	Q Inspection of homes	Electricity or gas (85%), paraffin/coal (15%)	- Parents: education, age - Smoking - Type of home - Agglomeration of people - Children: sex, birth weight, breastfeeding	RR (IC 95%) of ARI correlated to the use of electricity or gas versus paraffin or coal as a cooking fuel: All ARIs: 1.13 (0.84–1.52) Moderate/severe ARI: 0.65 (0.40–1.05)

LRTI: Lower respiratory tract infection; LRTI: lower respiratory tract infections; ARI: Acute respiratory infection; Q: questionnaire.

**Table 3:** Studies analyzing the relationship between the use of solid fuels, biomass, fuel oil, and kerosene in the home and the respiratory health of children under the age of 5.

Location Year Author	Design	Sample population: Age Number of cases	Disease studied		Exposure			Control variables	Results: adjusted OR/RR (IC 95%)
			Definition of the case	Information source	Source, chemical products	Evaluation	Levels (% exposure)		
							
Studies conducted in Africa and Asia									

2 suburbs of Delhi: Kusumpur Pahari and Kathpulty 1998 Sharma et al. [17]	Case-control prospective	633 children (320 <1year of age) - 316 Kathapulty - 317 Kusumpur	Acute lower respiratory tract infection (ALRTI)	Q	wood kerosene		Kathapulty: 45% W 55% K Kusumpur: 55% W 45% K	- Job, parents' educational level - Homes with >1bedroom, location of kitchen, - Family size - Siblings …	There were 3 children with three episodes of acute lower respiratory tract infections in the wood group as compared to the kerosene group, but in the latter, there was a tendency to cook inside the home in the presence of the children.

Northern India 2001 Broor et al. [18]	Case-control	512 children under the age of 5	Severe acute lower respiratory tract infection. Cases (201): admitted to hospital Controls (311): healthy children attended to in the same hospital's pediatric outpatient clinic.	Survey (parents)	Use of fuel other than liquid petroleum gas for cooking: biomass, wood, harvest waste, dung	Questionnaire (parents)	36.8% of the cases and 20.6% of the controls used a cooking fuel other than liquid petroleum gas.	- Parents: age, education - Smoking - Type of house: semi-attached with foundation - Children: breastfed, vaccinated	OR for ALRTI associated with the use of cooking fuel other than liquid petroleum gas. 2.51 (1.51–4.16)

Calcutta 2002 Mahalanabis et al. [19]	Case-control	2–35 months Cases: 127 Controls: 135	Pneumonia Cases: hospital admission with pneumonia Controls: from an outpatient immunization clinic	Hospital registers Hospital diagnosis of pneumonia	Use of solid fuel	Q		- Mother's education - Economic status (televisions, electric fans …) - Number of siblings, large animals - Child: nutrition, history of asthma	Use of solid fuel OR: 3.97 (2:00–7.88)

India 2005 Mishra [14]	Transversal	29,768 children 0–35 months	Acute respiratory infection (ARI) (coughing with tachypnea in the two weeks prior to the survey)	Children selected in a national health survey (National Family Health Survey NFHS-2)	(ídem)	Q	Highly pollutant	Covariables added: status of the head of family, type of house, separate kitchen or not, agglomeration of people (>3 people per room or >3).	- Highly pollutant fuels: 1.82 (1.58–2.09) - After adjusting for various covariables: 1.58 (1.28–1.95) - In homes where both biomass and cleaner fuels were used 1.41 (1.17–1.70) - Boys/girls: 1.12 (1.01–1.23)

Suburbs of Bangladesh (Dhaka) 2007 Khalequzzaman et al. [20]	Case-control	116 Children under the age of 5 65 biomass 51 solid fuel	*During the previous month * Prevalence of symptoms and signs: Reddening and itching of eyes, itchy skin, nasal congestion, cough, shortness of breath, intercostal muscle retraction, wheezing, sore throat Prevalence of illness: Respiratory problems, bronchiolitis, asthma, pneumonia Eczema, diarrhea, viral fever	Q and examination of symptoms	Indoor air concentrations of VOCs, CO, CO_2_, NO_2_ and dust particles.		Biomass 53% Fossil fuel 47%	- Mother's age, educational level and job - Smoking in the house by care-taker - Family income, cost of fuel -Ventilation	Use of biomass versus fossil fuels - Cough 1.9 (0.6–6.3) - Shortness of breath 6.3 (1.6–29.1) - Intercostal muscle retraction 5.5 (1.3–29.4); wheezing 4 (1.1–16.2) - Respiratory illness 2.5 (0.5–16.3)

									
Studies conducted in South America									

Guatemala (indigenous group) 2004 Schei et al. [21]	Transversal	1058 children between 4–6 years of age	- Asthma diagnosed - Occasional wheezing in the last 12 months, induced by exercise in the last year, no. of episodes in the last year (>4*) - Night cough in the last year - Alterations in sleeping habits - Speech limitations - All severe criteria.	Q (ISAAC)	Use of wood for cooking fuel	(1) cooker with exhaust chimney (2) mix of gas and open fire cooking (3) open fire cooking	(1) 51.5% (2) 10.4% (3) 38.1%		OR for open fire cooking wheezing: - Occasional: 2.0 (1.1–3.7) - Last 12 m: 3.4 (1.3–8.5) - Induced by exercise in the past year: 3.5 (1.4–8.6) - Speech limitations: 3.4 (1.1–11.3)

Studies conducted in the United States									

Connecticut and Virginia 2002 Triche et al. [9]	Birth cohort obtained in a hospital	890 Children 1 year of age	Days of wheezing Episodes of wheezing Days of cough Episodes of cough	Q/2 weeks	- Wood-burning heater - Kerosene heater - Gas heater	Q	24.6% 18.1% 17.4%	- Socio-demographics - Mother: allergies, asthma, education - No. of children - Child: breastfed, sex, birth season	**- **Total days of cough Wood-burning heater: 1.10 (1.02–1.19) - Episodes of cough Kerosene heater: 1.07 (1.00–1.15) - Other correlations: NS

Arizona 1996 Robin et al. [22]	Case-control 1 : 1 pairing	1–24 months (mean = 7) 45 cases and 45 matched controls.	Children hospitalized with acute lower respiratory tract infections. Controls: children with health record at the same hospital matched 1 : 1 on date of birth and gender.		Energy sources for heating and cooking		Gas/electric: Mean = 22.2 MG = 18,8 + wood heating: M = 100.9 MG = 57.4 Only wood: M = 85.6 GM = 62.8	Running water, type of house, no. of rooms, no. of children, distance from clinic/hospital, passive exposure to tobacco smoke.	A risk 5 times greater was found for children who lived in houses in which wood was used for cooking, albeit with an ample interval of confidence due to the low number of subjects in this group (8.9% of the cases). The magnitude of correlation decreased less than 20% after adjusting for the potentially confounding variables.

Studies conducted in Europe									

Finland 2001 Kilpelainen et al. [23]	Cohorts (retrospective)	- Survey administered to 10,667 university students (18–25years) about their first 6 years of life - (children born in the mid-70s)		Q	Gas heaters	Q	51.7% Farm 19.4% Rural, not farm 28.9% Urban	- Parents: history of asthma or atopy - No. of children, pets with hair or feathers, family member who smoked indoors daily, daycare outside the home for children under 2, sex of child	- A correlation was found between living on a farm between the ages of 0-6 and allergic rhinoconjunctivitis 0.61 (0.5–0.75; *P* < .0001). Living on a farm was frequent for subjects whose homes were heated with wood.

Teplice (Czech Rep.) 2006 Baker et al. [24]	Cohort	452 children followed up to the age of three	Lower respiratory tract illness (except pneumonia). ICD-10 J20, J21, J40 and J44. 1049 episodes (98% acute bronchitis J20)	- Registers from pediatric practices, emergency rooms, and hospitals. - Q (mothers)	Heating fuels (natural gas, electricity, coal, or wood) and cooking fuels (gas, propane, electricity, coal, or wood)	Q	?	- Parents: age, education, ethnic background of mother. Father's education and smoking - Home: temperature and humidity - Family density, other children <14 years of age - Child: Sex, breastfed	- Coal stoves - RR 1.45 (1.07–1.97) - With breastfeeding: RR 1.33 (0.95–1.86) - Without breastfeeding: RR 2.77 (1.45–5.27) - Remaining sources NS

ALRTI: acute lower respiratory tract infections; Q: questionnaire; W: wood; K: kerosene; VOCs: Volatile organic compounds; CO: Carbon Monoxide; CO_2_: Carbon Dioxide; NO_2_: Nitrogen Dioxide; NS: not statistically significant.

**Table 4:** Studies analyzing the relation between chemical compound sources in the home and respiratory health in children under 5 years of age.

Location Year Author	Design	Sample population: Age Number of cases	Disease studied		Exposure			Control variables	Results: adjusted OR/RR (IC 95%)
			Case definition	Information source	Source of chemical products	Evaluation	Levels (% exposure)		
							
Studies conducted in Europe									

Oslo, Norway 1999 Jaakkola et al. [27]	Case-control nested in a birth cohort	2 first years of life 251 pairs	Case: ≥2 episodes of bronchial obstruction or 1 episode ≥4 weeks	- Q, at birth and at 6, 12, 18, and 24 months. - Reports from the family physician - Medical registers	Floor and wall covering materials	Home visit with systematic inspection	Floors PVC: Ca 72%, Co 59% Carpeting Ca 39%, Co 46% Walls Paper with PVC Ca 32% Co 34% Cloth wallpaper Ca 31%, Co 22%	- Atopy in parents - Problems with humidity and other variables related to the surface covering materials used in the home - Passive exposure to tobacco smoke - Siblings - Day care, breastfeeding, sex	Floors: - PVC in 1 or more rooms: 1.89 (1.14–3.14) - Carpeting in 1 or more rooms: 0.74 (0.48–1.13) Wall coverings: - Paper with PVC in 1 or more rooms: 0.72 (0.42–1.22) - Cloth wallpaper in 1 or more rooms: 1.58 (0.98–2.54)

Oslo, Norway 1999 Oie et al. [28]	Paired case-control within a birth cohort	2 first years of life - 304 pairs - Study of ventilation carried out for 172 pairs	Case: ≥2 episodes of bronchial obstruction or 1 episode ≥4 weeks	Q: at birth and at 6, 12, 18, and 24 months. Reports from the family physician Medical registers	Textile or plastic indoor surfaces Ventilation of the home	Q, home visit with systematic inspection and measurement of ventilation	37% of the homes were insufficiently ventilated	- History of atopy in parents - Problems with humidity - Passive exposure to tobacco smoke - Siblings, breastfeeding, daycare at age one, sex	Cloth wallpaper All children: 1.7 (0.94–2.93) *Depending on the ventilation of the home: * - Low: 3.7 (0.62–21,53) - High: 1.7 (0.72–3.94) - Index of exposure to plastics: All: 2.9 (1.54–5.49) *Depending on the ventilation of the home: * - Low: 12.6 (1.00–159) - High: 2.6 (1.02–6.58)

Espoo, (Helsinki) Finland 2000 Jaakkola et al. [29]	Transversal	1–7 years n: 2568	- Current asthma - Allergic rhinitis - Persistent wheezing - Persistent cough - Persistent phlegm - Congestion/nasal secretions - Respiratory infections	Q	Plastic wall coverings	Q	Plastic wall coverings in 2.8% of the homes	- Parents' education, a sole caregiver - Presence of pets with fur or feathers, mold, and humidity - Passive exposure to tobacco smoke, - Daycare outside the home, sex, age	Persistent wheezing: 3.42 (1.13–10.36) Persistent cough: 2.41 (1.04–5.63) Persistent phlegm: 2.76 (1.03–7.41) For the rest of the indicators, the results were not statistically significant

Leizpig, Germany 2000 Diez et al. [30]	Birth cohort (high risk: premature or with risk factors for allergies)	Follow-up: Check up at 6 weeks (n: 339) At l year (310)	Respiratory infections (6 weeks) Wheezing (1 year)	Q (parents) Clinical check up	Redecorating during pregnancy	Q	Redecorating in 67% of the homes: 37% new carpeting 17% new adhesive carpeting 48% new paint 52% new furniture	- Heating, gas for cooking - Size of home - Pets	OR - Respiratory infections in children at 6 weeks of age: Redecorating: 5.6 (1.3–24.0) - Wheezing in children 1 year of age: Redecorating: 1.9 (1.1–3.5)

Leizpig, Germany 2003 Diez et al. [31]	Birth cohort (high risk)	First 2 years of life n: 186	Respiratory symptoms: wheezing, panting, obstructive bronchitis (excluding laryngitis)	Q (parents)	Redecorating in the home	Q	During pregnancy: 66% During the first year: 19% During the second year: 19%	- Atopy in parents, - Humidity, pets - Tobacco smoke - IgE, birth weight, sex	OR - During the first year: Obstructive bronchitis: 4.1 (1.4–11.9) Wheezing: 2.4 (0.7–8.6) - During the second year: Obstructive bronchitis: 4.1 (1.4–12.9) Wheezing: 3.0 (1.0–9.1)

Stockholm 2004 Emenius et al. [32]	Paired case-control from the BAMSE cohort	From birth to the age of 2 181 cases 359 controls paired by age	Recurrent wheezing	Q (parents)	Having painted the home/child's room in the year prior to the child's birth or during the first year of life. Ventilation in the home	Q, home visit with systematic inspection and measurement of ventilation	Painting the house but not the child's room: Cases (16.6%) Controls (20.9%) Painting the child's room: Cases (40.9%) Controls (30.6%)	- Maternal age, parents' medical history (asthma, allergies), maternal smoking - Age of building - Breastfeeding	Painting the house but not the child's room: 1.1 (0.7–1.9) Painting the child's room: 1.7 (1.1–2.6) The amount of ventilation did not modify the correlation

Q: questionnaire
